# Inhibition of breast cancer cells by targeting E2F-1 gene and expressing IL15 oncolytic adenovirus

**DOI:** 10.1042/BSR20190384

**Published:** 2019-07-23

**Authors:** Yang Yan, Hu Xu, Jiandong Wang, Xin Wu, Wei Wen, Yan Liang, Lingdi Wang, Fengyuan Liu, Xiaohui Du

**Affiliations:** 1 Department of General Surgery, Chinese People’s Liberation Army General Hospital, Beijing 100853, China; 2 Department of General Surgery, Hainan Hospital of Chinese People’s Liberation Army General Hospital, Sanya 572013, China; 3 Department of Pediatrics, Chinese People’s Liberation Army General Hospital, Beijing 100853, China

**Keywords:** breast cancers, E2F-1, IL-15, Oncolytic adenovirus

## Abstract

The wide application of oncolytic adenovirus presents a novel therapeutic strategy for breast cancer gene therapy. Application of adenovirus alone achieves little curative effects on breast cancer. In addition, it is worth exploring the synergistic anti-tumor effect by inserting immunomodulatory factor in oncolytic adenovirus genome. By taking the advantage of the highly proliferative property of breast cancer, a novel recombinant adenovirus which could selectively kill tumor cells is established under an E2F-1 promoter. Also by carrying human Interleukin-15 (IL-15) gene, the oncolytic adenovirus exhibits an immunomodulatory effect. The present study proved that the novel oncolytic virus (SG400-E2F/IL-15) exhibits an enhanced anti-tumor activity both *in vitro* and *in vivo*, representing an experimental basis for breast cancer “virus-gene” therapy.

## Introduction

Breast cancer is well-known as the most common malignant tumor in women [1]. Despite the improvement of survival rate due to comprehensive surgical treatment modality, it is still a major challenge treating patients with advanced or recurrent breast cancer [2]. As a new treatment technique, oncolytic adenovirus (a conditionally replicative adenovirus) provides a novel therapeutic modality for breast cancer with its appropriate targeting property [3–5].

To obtain controlled replication of adenovirus, the gene required for adenovirus growth is usually placed under the control of a tumor-specific promoter or enhancer [6]. The transcriptional factor E2F-1 plays a significant role in the control of cell cycle, proliferation, and carcinogenesis; a higher expression of E2F-1 is often detected in breast cancer tissues compared with normal tissues [7,8], suggesting that E2F-1 may be an effective target for treatment of breast cancer with oncolytic virus. Replacement of the endogenous promoter of adenovirus type 5 with the E2F-1 promoter may construct the recombinant adenovirus highly expressed E2F-1 gene in tumor tissues [9,10].

In order to improve the therapeutic effect, an immune regulatory factor is often inserted to the oncolytic virus vector genome to exert the dual curative effect (virus treatment and gene therapy) with a long-term treatment efficacy [6]. Interleukin-15 (IL-15) is a crucial cytokine, enhancing the activity of macrophages and neutrophils, accelerates the production and activation of effector T cells (especially natural killer [NK] cells, cytotoxic [CD8^+^] T cells, and natural killer T [NKT] cells), and regulates the survival and proliferation of memory T cells. Recently, IL-15 has been shown to inhibit the proliferation of breast cancer cells via various immune cells [11,12]. However, IL-15 has not been applied on breast cancer gene therapy in spite of its high potential anti-tumor property.

In the present study, we attempted to investigate an association between the clinical data and E2F-1 expression in breast cancer. In addition, a novel recombinant adenovirus has been constructed that targeted E2F-1 and expressed IL-15, and validated its inhibitive effect on breast cancer cells, providing new experimental basis for breast cancer gene therapy.

## Materials and methods

### Patients and specimens

A total of 85 tissue samples were collected from breast cancer patients undertaken radical mastectomy with pathologically confirmed invasive ductal carcinoma (IDC) at the People’s Liberation Army (PLA) General Hospital since January 2016 to October 2016. All patients received no radio- or chemo-therapy before surgery, while those with incomplete information were excluded.

### Histological examination

Protocols for histological examination (HE) staining and immunohistochemistry (IHC) were implemented as previously described [13]. Deparaffinized tissue sections were treated with anti-human E2F-1 monoclonal antibody (Abcam). After incubation with the appropriate secondary antibody system (Thermo), the proteins were detected using 3,3-diaminobenzidine (Sigma) enhancement. The slides were counterstained with hematoxylin. Anti-IL-15 monoclonal antibody (Abcam) was utilized to detect the expression of IL-15.

### Western blot

Total protein samples from tumor tissues were extracted using RIPA buffer and cells from the plates were resuspended in lysis uffer. Then the protein concentration of each sample was determined using the Bradford method (Thermo). Equal amounts of protein (100 μg) were separated via SDS/PAGE and transferred to a PVDF membrane (Millipore). The membrane was blocked in 8% non-fat dry milk for 1 h at 37°C prior to incubation in the primary antibody (Santa Cruz) at room temperature for 2 h. After washing with Tris-buffered saline, the membrane was incubated in anti-rabbit or antimouse IgG (Santa Cruz) for 1 h at room temperature. The blots were visualized via enhanced chemiluminescence (ECL) according to the manufacturer’s protocol. The primary antibodies of E2F-1, E1A, GAPDH, and IL-15 were purchased from Santa Cruz Biotechnology.

### Cells and cell culture

Human embryonic kidney cell lines HEK293 and 293T, human breast cancer cell line MDA-MB-231 and human gastric cancer cell line BGC823 were purchased from the Cell Center of Peking Union Medical College. MDA-MB-231-luc cells that stably express firefly luciferase gene were purchased from Cell Biolabs company. Human lung fibroblast cells (MRC-5) and human colorectal cancer cell line SW480 were purchased from Shanghai Institute of Biochemistry and Cell Biology, Chinese Academy of Sciences. MDA-MB-231 and MDA-MB-231-luc cells were cultured in Leibovitz’s L-15 medium containing 10% fetal bovine serum (FBS), at the temperature of 37°C in CO_2_-free conditions. HEK293 and MRC-5 cells were maintained in Eagle’s minimal essential medium (EMEM) supplemented with 10% FBS, at the temperature of 37°C, and 5% CO_2._ The other cell lines were cultured in Dulbecco’s modified essential medium (DMEM) supplemented with 10% FBS, 4 mM glutamine, 50 U/ml penicillin, and 50 mg/ml streptomycin at the temperature of 37°C, and 5% CO_2_.

### Plasmids and viruses

The virus skeleton plasmid PPE3, PXC20 plasmid (containing the E1 gene), P55-E2Fp plasmid (containing the regulatory sequence of the E2F-1 promoter), and PENTER12-hIL2SPIL15 plasmid (carrying IL-15 gene) were constructed at the laboratory of the Eastern Hepatobiliary Surgery Hospital (Shanghai, China). The recombinant adenovirus was constructed as previously described [13,14]. In brief, P55-E2Fp and PXC20 vectors were digested by *Xho*I and *EcoR*I, positive clones were selected, and plasmid DNA was extracted and verified with restriction enzyme digestion, which termed as PXC20-E2Fp. PXC20-E2Fp and PPE3 were co-transfected into 293 cells with Lipofectamine 2000 (Gibco-BRL). The purified and validated adenovirus was named SG400-E2F. Plasmids PXC20-E2Fp and PPE3-hIL2SPIL15 were co-transfected into 293 cells, and the verified recombinant adenovirus was called SG400-E2F/IL-15. Moreover, replication deficient adenovirus SG400-EGFP, which expressed as green fluorescence protein, was used to control. In addition, median tissue culture infective dose method (TCID50) was utilized to determine the virus titer.

### Fluorescence microscopy

MDA-MB-231 and MRC-5 cells were infected with SG400-EGFP at a multiplicity of infection (MOI) of 1, and were observed under the TE2000-U fluorescence microscope (Nikon) [15]. Photographs were taken at 24, 48, and 72 h after infection.

### Cell growth inhibition assay

MDA-MB-231 and MRC-5 cells (1 × 10^4^ cells) were seeded on 96-well plates. After passing 18 h, SG400-E2F, SG400-E2F/IL-15, and SG400-EGFP were added at different MOI values (1000, 100, 10, 1, and 0.1 pfu/cell). After passing 2 h, the media were removed and placed with fresh media (100 μl) containing 5% FBS. At the fifth day, 3-(4,5-Dimethylthiazol-2-yl)-2,5-diphenyltetrazolium bromide (MTT, Sigma–Aldrich) (100 μl) were added and incubated at temperature of 37°C for 4 h. After removal of the supernatants, dimethyl sulfoxide (DMSO) (100 μl) was added and the absorbance value was measured at 490 nm in order to draw the cell growth curve.

### Cell cycle analysis

As described previously [13], the cellular DNA content was analyzed using the Cycle TEST™ PLUS DNA Reagent Kit (Becton Dickinson) according to the manufacturer’s protocol.

### Detection of IL-15 protein in culture supernatants and cells

MDA-MB-231 and MRC-5 cells were seeded on six-well plates at 3 × 10^5^/well. After 24 h, cells were infected with adenoviruses SG400-E2F1/IL-15 (MOI = 5) for 2 h. Then, virus particles were collected at 12, 24, 48, and 96 h post-infection. IL-15 in the supernatants was detected by an ELISA (enzyme-linked immunosorbent assay) kit (R&D Systems) [16]. Furthermore, cells were collected and the protein expression of IL-15 was measured by Western blot.

### Establishment of *in vivo* animal model

Six-week old female BALB/c nude mice weighed 19–21 g were purchased from the Animal Center of Peking Union Medical College. Protocols were approved by the Animal Ethics Committee of Chinese PLA medical school and the Guidelines of Animal Ethics of Ministry of Science and Technology. MDA-MB-231-luc cells were diluted to 8 × 10^7^/ml in PBS and mixed with matrigel (1:1). BALB/c nude mice were anesthetized and injected in the mammary fat pat with 50 μl of tumor cells. When tumors reached a volume of 100 mm^3^, 20 mice were randomly divided into four groups and injected with an equal volume (50 μl) of PBS, SG400-EGFP, SG400-E2F, and SG400-E2F/IL-15 (1 × 10^10^ pfu/ml). Injection was repeated twice per day for a total of three times. Tumor volume was weekly measured and the growth curve was drawn. Tumors were measured using *in vivo* stereo imaging system (IVIS 200, Xenogen Corporation) weekly [15]. Eventually, tumors were collected and fixed in 10% formalin after mice were euthanized.

### Statistical analysis

The χ^2^ test was carried out in order to examine the various clinicopathological characteristics with the expression of E2F-1. The difference between those four groups was analyzed by analysis of variance (ANOVA) and Student’s *t*-test using SPSS statistical software (Version 19.0) (SPSS Inc.) as well as Prism 5 software (GraphPad). In addition, *P*-value < 0.05 was statistically considered significant.

## Results

### Expression of E2F-1 in human breast cancer tissues

**Figure 1:**
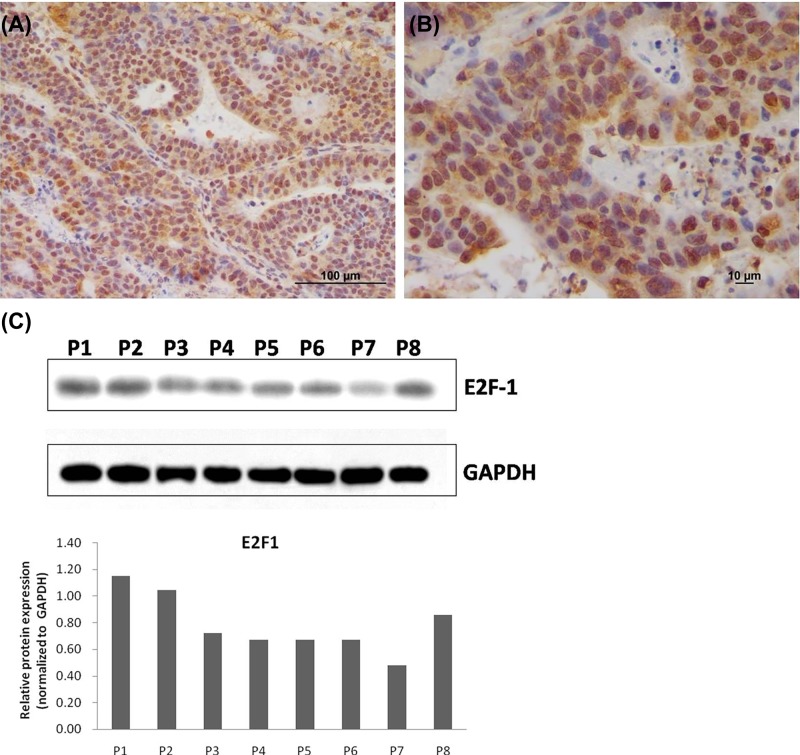
Expression of E2F-1 in human breast cancer tissue Immuohistochemistry was performed to detect the expression of E2F-1 in breast cancer tissues. Original magnification (**A**) ×200 (**B**) ×400 (**C**) Western blot analysis of E2F-1 gene expression in IDC tumor tissues from eight different patients.

The results showed that E2F-1 was positively expressed in 45 out of 85 cases (52.9%) of IDC based on IHC result (Figure 1). After analyzing the available association between E2F-1 and clinical data, it was revealed that E2F-1 expression was associated with tumor lymph node metastasis, and Tumor Node Metastasis (TNM) system, but not with age and tumor volume (Table 1). These achieved data demonstrated that E2F-1 may serve as a potential target for breast cancer gene therapy. Thus, in the present study, a recombinant oncolytic adenovirus was constructed targeting E2F-1 for further investigations.

**Table 1:** Correlation between E2F-1 expression and clinicopathological features in IDCs

Clinicopathologic features	E2F-1		*P*-value
	Positive (%)	Negative (%)	
Ages (years)			0.600
≤50	25 (56.8)	19 (43.2)	
>50	20 (48.8)	21 (51.2)	
Tumor size (diameter)			0.235
≤2cm	19 (45.2)	23 (54.8)	
>2 cm	26 (60.5)	17 (39.5)	
Clinical stage			0.004
I	6 (23.1)	20 (76.9)	
II	14 (66.7)	7 (33.3)	
III	16 (66.7)	8 (33.3)	
IV	9 (64.3)	5 (35.7)	
Lymph node metastasis			0.015
Negative	21(41.2)	30 (58.8)	
positive	24 (70.6)	10 (29.4)	

*P*<0.05, statistically significant

### Oncolytic activity of SG400-E2F/IL15 *in vitro*

**Figure 2:**
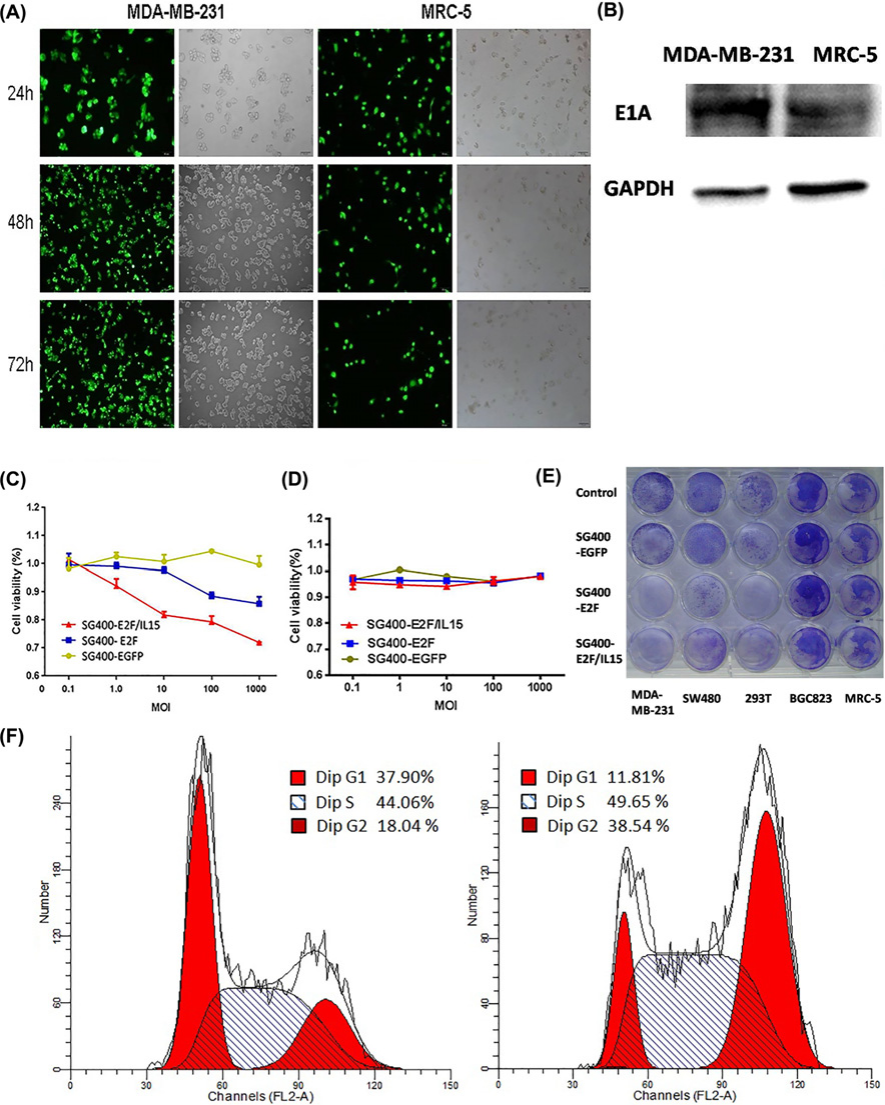
SG400-E2F/IL-15 selectively inhibited breast cancer cell proliferation (**A**) Representative photomicrographs were obtained from MDA-MB-231and MRC-5 infected with SG400-EGFP at the MOI of 1. Original magnification, 200×. (**B**) The expression of adenovirus structure protein E1A in MDA-MB-231 and MRC-5 cells 48 h after infection of SG400-E2F/IL15. 5 days after infection with SG400-E2F/IL15, MTT assay was performed to measure the proliferation of MDA-MB-231 (**C**) and MRC-5 (**D**) at different MOI (Panel C: SG400-EGFP and SG400-E2F, *P*=0.0002; SG400-E2F and SG400-E2F/IL15 *P*=0.0000. Panel D: SG400-EGFP and SG400-E2F, *P*=0.838; SG400-E2F and SG400-E2F/IL15, *P*=0.356). (**E**) Different cell lines (1 × 10^5^ cells/well) were cultured in 24-well plates and treated with SG400-EGFP, SG400-E2F, or SG400-E2F/IL15 (5 MOI) with PBS used as control. 3 days after infection, cytotoxicity was observed via crystal violet staining. (**F**) Cell cycle changed in MDA-MB-231 cells 5 days after infected with SG400-E2F/IL15 (10 MOI). Left panel: Uninfected MDA-MB-231 cells. Right panel: MDA-MB-231 cells infected with SG400-E2F/IL15.

The endogenous promoter of adenovirus type 5 was replaced with E2F-1 promoter to obtain SG400-E2F (8.5 × 10^10^ pfu/ml). Meanwhile, the virus SG400-E2F/IL-15 contained regions encoding human IL-15 (1.8 × 10^10^ pfu/ml). Moreover, E1A-deficient SG400-EGFP with cytomegalovirus (CMV) promoter was used as control (5 × 10^10^ pfu/ml). MDA-MB-231 and MRC-5 cells were infected with SG400-EGFP and observed using a fluorescence microscope as displayed in Figure 2A. It was revealed that adenovirus type 5 was able to infect both tumor cells and normal fibroblasts, and effectively expressed green fluorescent protein (GFP). The human E2F-1 promoter was operably linked to E1A in SG400-E2F1 and SG400-E2F/IL-15 to restrict expression of E1A to Rb pathway-defective tumor cells. We used Western blot to demonstrate the plasmids was expressed in MDA-MB-231 cells and MRC-5 cells (Figure 2B). 48 h after infection, adenovirus structure protein E1A were founded in both MDA-MB-231 cells and MRC-5 cells, but MDA-MB-231 cells had higher expression level.

Compared with SG400-EGFP, infection with SG400-E2F1 and SG400-E2F/IL-15 both inhibited MDA-MB-231 cell growth in a dose-dependent manner (Figure 2C); SG400-E2F/IL-15 exhibited a stronger inhibitive effect than SG400-E2F1 (*P*=0.0000). However, the recombinant virus had no significant effects on MRC-5 cell growth as shown in Figure 2D.

3 days after infection (5 MOI), cells were stained with crystal violet. For cell lines with a high expression level of the E2F-1 gene such as MDA-MB-231 and SW480, the amount of residual cells after treatment with SG400-E2F1 and SG400-E2F/IL-15 was less than that in the MRC-5 group (Figure 2E). 5 days after infected with different adenovirus (10 MOI), residual MDA-MB-231 cells were analyzed by flow cytometry. Relative to normal MDA-MB-231 cells (G1: 37.9%; S: 44.06%) and SG400-EGFP (G1: 35.28%; S: 42.93%), SG400-E2F1 (G1: 18.57%; S: 51.93%) and SG400-E2F/IL15 (G1: 11.81%; S: 49.65%) treatment produced lower percentages of cells in G1 phase but significantly higher percentages of cells in S phase (Figure 2F).

**Figure 3:**
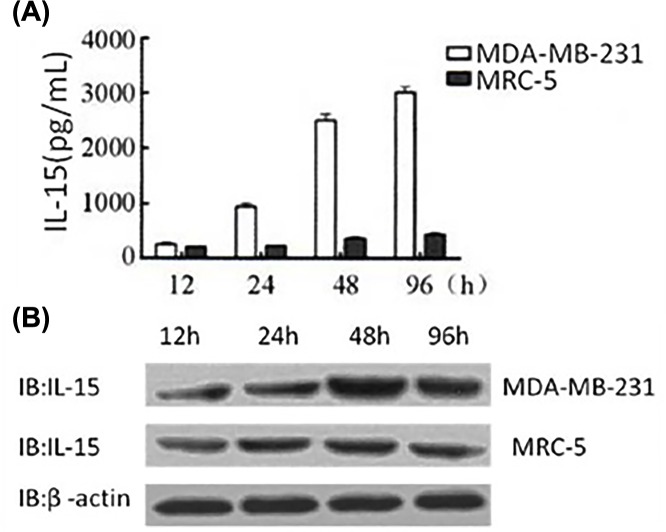
Expression of IL15 in MDA-MB-231 and MRC-5 cells (**A**) ELISA was used to detect the production of IL15 in the supernatants of MDA-MB-231 and MRC-5 cells infected with SG400-E2F/IL15. (**B**) Western blotting was performed to detect the expression of IL15 in MDA-MB-231 and MRC-5 cells after infection with SG400-E2F/IL15.

24 h after infection with SG400-E2F/IL-15, the secretion of IL-15 from MDA-MB-231 cells was enhanced. Concentration of IL-15 reached 3ng/ml at 96 h after infection of MDA-MB-231, while low expression levels of IL-15 remained in MRC-5 cells (Figure 3A). Similarly, the protein expression of IL-15 in tumor cell lysates increased in a time-dependent manner, while a low IL-15 level maintained in normal fibroblasts as illustrated in Figure 3B.

### Oncolytic activity of SG400-E2F/IL15 *in vivo*

**Figure 4:**
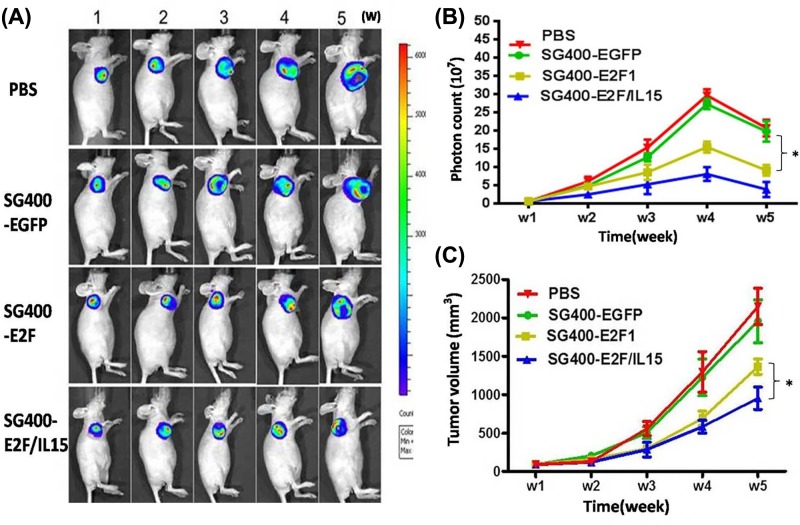
Inhibitive effects of SG400-E2F/IL15 in a mouse model of breast cancer A mouse model of breast cancer was established after inoculation with MDA-MB-231-luc cells. Mice were randomly divided into four groups and injected with PBS, SG400-EGFP, SG400-E2F, and SG400-E2F/IL15. Fluorescence imaging (**A**) photon counting (**B**) and tumor volume measurement (**C**) were performed weekly. The data were presented as mean ± SD (*n*=5 per group, ^*^P<0.05).

After establishment of tumor xenografts, mice were injected with PBS, SG400-EGFP, SG400-E2F1, and SG400-E2F/IL-15. Additionally, luminescence imaging (Figure 4A), photon counting (Figure 4B), and tumor volume measurements (Figure 4C) were performed. Consequently, it was disclosed that the number of photons was higher in PBS and SG400-EGFP groups than SG400-E2F and SG400-E2F/IL-15 groups (*P*<0.05) as depicted in Figure 4B. The present study revealed that SG400-E2F/IL-15 had a strong inhibitive effect on tumors compared with SG400-EGFP and PBS groups (Figure 4C) (SG400-E2F/IL-15 vs. PBS, *P*=0.009; SG400-E2F/IL-15 vs. SG400-EGFP, *P*=0.012; SG400-E2F/IL-15 vs. SG400-E2F1, *P*=0.035).

**Figure 5:**
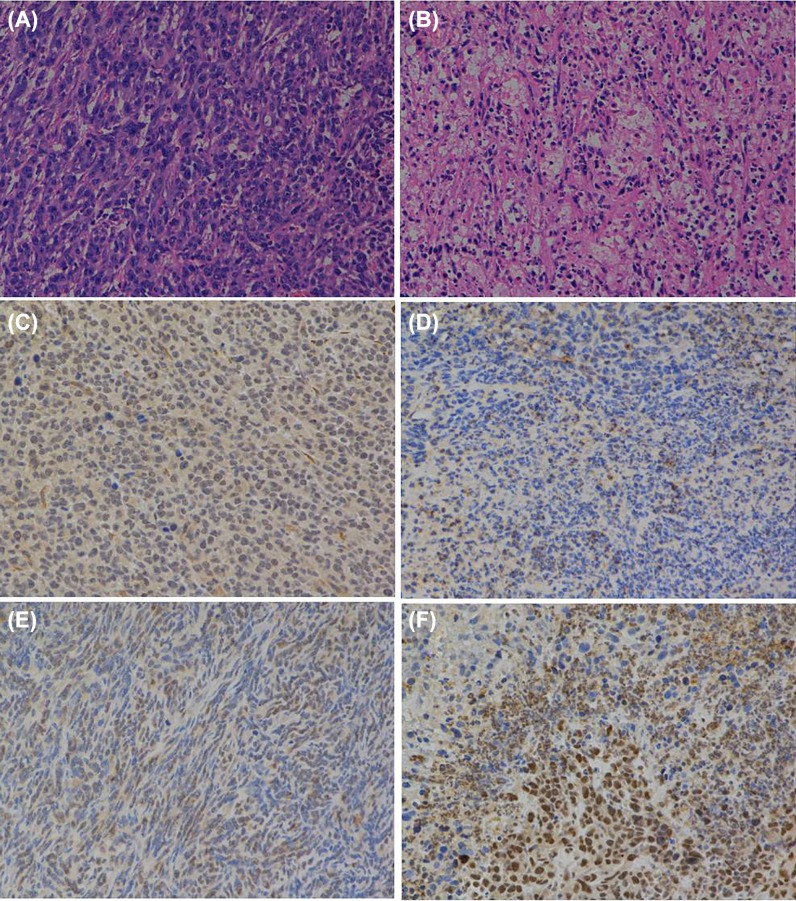
Pathological and immunohistochemical examination after infection with SG400-E2F/IL15 HE staining of tumor tissues in PBS group (**A**) and SG400-E2F/IL15 group (**B**). Immunohistochemical analysis of E2F-1 (**C,D**) and IL-15 (**E,F**) of tumor tissues in PBS and SG400-E2F/IL15 group. Magnification 200×.

*HE* staining, as shown in Figure 5A,B, unveiled more necrotic tissues in SG400-E2F/IL-15 group than SG400-E2F group, demonstrating different *in vivo* inhibitive effects of adenoviruses on the proliferation of breast cancer cells. Immunohistochemistry analysis showed an organized tumor tissue and highly expressed E2F1 in PBS and SG400-EGFP injected mice. Meanwhile, mice in the SG400-E2F group exhibited partly necrotic tissues and disorganized tissues. Furthermore, SG400-E2F/IL-15 injection triggered cell rupture in E2F1-positive tumor cells as well as a large amount of tissue necrosis (Figure 5C,D). Compared with the PBS group, an elevated expression of IL-15 was observed in mice injected with SG400-E2F/IL-15 as illustrated in Figure 5E,F.

## Discussion

Breast cancer is a common malignant tumor in women. Several factors are involved in the occurrence and development of breast cancer. Among those factors, cell cycle-related genes play a crucial role in tumor cell growth. E2F1, a member of the transcription factor family E2F, regulates the transition of cell cycle from G1 phase to S phase. Activation of E2F1 contributes to the expression of genes involved in DNA synthesis and promotes cell growth [7]. Additionally, E2F1 is highly expressed in breast cancer, contributing tumor cell proliferation and predicts poor prognosis [17,18]. Similarly, the present study revealed that E2F1 was up-regulated in patients with tumor lymph node metastasis and advanced stage (see Table 1). Thus, further investigations should be performed to validate the potential application of E2F1 on breast cancer gene therapy.

Oncolytic adenovirus alone exhibits little anti-tumor property [19] probably due to the antiviral immunity and immune escape in tumor microenvironment. In most studies, the immunomodulatory elements are typically inserted to the oncolytic virus genome (i.e., immune-enhancing oncolytic virus). Therefore, the recombinant virus can directly kill tumor cells, accordingly, release immune regulatory factors. Thus, the immunomodulatory ability and the synergistic anti-tumor effect were enhanced to achieve a long-term treatment efficacy. Currently, the co-stimulatory molecule granulocyte macrophage–colony stimulating factor (GM–CSF) is the most widely used factor loaded to the oncolytic virus. GM–CSF can promote dendritic cell (DC) maturation, improve its antigen-presenting ability, thereby enhancing the specificity of anti-tumor ability. In terms of breast cancer, it has been reported that oncolytic adenovirus carrying IL-24 exhibits obvious anti-tumor effect in an animal model of breast cancer [20]. In the present study, ELISA and Western blotting analysis showed that SG400-E2F/IL-15 infection increased the expression of IL-15 in both supernatants and lysates of MDA-MB-231 cells. In addition, *in vivo* assay also revealed elevated IL-15 levels in tumor tissues after infecting with SG400-E2F/IL-15. The achieved data demonstrated that the novel oncolytic virus conforms to the requirement of constructing immunity-enhanced oncolytic virus. Moreover, this novel oncolytic virus selectively kills tumor cells and simultaneously releases IL-15, as well as enhancing the inhibitory effect on breast cancer, even on triple negative breast cancer which tests negative for estrogen receptors, progesterone receptors, and excess HER2 protein [21].

In spite of wide infection ability of adenovirus type 5 on tumor cells and normal non-malignant cells, MTT and *in vivo* assays showed that SG400-E2F/IL-15 and SG400-E2F both could selectively infect and kill breast cancer cells; and SG400-E2F/IL15 exhibited highest cytocidal effects on tumor cells. In general, pRb/E2F pathway defects are widely present in solid tumors. Through the oncolytic effect of the virus, different kinds of self-derived tumor antigens can be obtained that are not limited by their subcellular localization, which is helpful to produce tumor-specific immune response with personalized and universal therapeutic significance. The replication of the virus leads to the expression of IL-15, and the high concentration of IL-15 can be obtained locally from the virus-infected tumor cells, which is favorable for stimulating the activity of immune cells, activating the systemic immune response, as well as enhancing the anti-tumor effect. These findings revealed that the novel adenovirus may have a wide clinical application on breast cancer “virus-gene” therapy.

## Abbreviations

ELISA: enzyme-linked immunosorbent assay
FBS: fetal bovine serum
GM–CSF: granulocyte macrophage–colony stimulating factor
HE: histological examination
IDC: invasive ductal carcinoma
IHC: immunohistochemistry
IL-15: interleukin-15
MOI: multiplicity of infection
PLA: People’s Liberation Army
