# Correction: Inhibition of breast cancer cells by targeting E2F-1 gene and expressing IL15 oncolytic adenovirus

**DOI:** 10.1042/BSR-20190384_COR

**Published:** 2020-07-21

**Keywords:** Oncolytic adenovirus, breast cancers, IL-15, E2F-1

Some substantive corrections are to be made to the original article “Inhibition of breast cancer cells by targeting E2F-1 gene and expressing IL15 oncolytic adenovirus” (*Biosci Rep.* (2019) **39**(7), DOI: 10.1042/BSR20190384). In Figure 2A, the photos of ‘72 hours after infection’ in the MDA-MB-231 group and ‘24 hours after infection’ in the MRC-5 group are replaced with the correct pictures in this correction article. This change does not affect the conclusion of this article.

**Figure 2:**
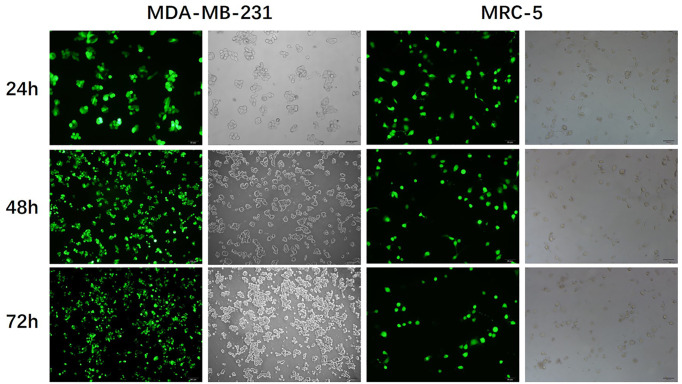
SG400-E2F/IL-15 selectively inhibited breast cancer cell proliferation (**A**) Representative photomicrographs were obtained from MDA-MB-231and MRC-5 infected with SG400-EGFP at the MOI of 1. Original magnification, 200×.

